# Effect of community health worker home visits on antenatal care and institutional delivery: an analysis of secondary outcomes from a cluster randomised trial in Mali

**DOI:** 10.1136/bmjgh-2022-011071

**Published:** 2023-03-22

**Authors:** Kassoum Kayentao, Rakesh Ghosh, Lamine Guindo, Caroline Whidden, Emily Treleaven, Calvin Chiu, Diego Lassala, Mohamed Bana Traoré, Jessica Beckerman, Djoumé Diakité, Aly Tembely, Ben Moulaye Idriss, Mohamed Berthé, Jenny X Liu, Ari Johnson

**Affiliations:** 1 Malaria Research & Training Centre, University of Sciences Techniques and Technologies, Bamako, Mali; 2 Route de 501 Lodgements SEMA, MUSO, Bamako, Mali; 3 Institute for Global Health Sciences, University of California San Francisco, San Francisco, California, USA; 4 London School of Hygiene & Tropical Medicine, London, UK; 5 Institute for Social Research, University of Michigan, Ann Arbor, Michigan, USA; 6 School of Public Health, University of California, Berkeley, California, USA; 7 District Sanitaire de Bankass, Mopti, Mali; 8 Ministère de la Santé et du Dévelopement Social, Bamako, Mali; 9 Institute for Health and Aging, Bixby Center for Global Reproductive Health, University of California San Francisco, San Francisco, California, USA

**Keywords:** maternal health, public health

## Abstract

**Introduction:**

Though community health workers (CHWs) have improved access to antenatal care (ANC) and institutional delivery in different settings, it is unclear what package and delivery strategy maximises impact.

**Methods:**

This study reports a secondary aim of the Proactive Community Case Management cluster randomised trial, conducted between December 2016 and April 2020 in Mali. It evaluated whether proactive home visits can improve ANC access at a population level compared with passive site-based care. 137 unique village clusters, covering the entire study area, were stratified by health catchment area and distance to the nearest primary health centre. Within each stratum, clusters were randomly assigned to intervention or control arm. CHWs in intervention clusters proactively visited all homes to provide care. In the control clusters, CHWs provided the same services at their fixed community health post to care-seeking patients. Pregnant women 15–49 years old were enrolled in a series of community-based and facility-based visits. We analysed individual-level annual survey data from baseline and 24-month and 36-month follow-up for the secondary outcomes of ANC and institutional delivery, complemented with CHW monitoring data during the trial period. We compared outcomes between: (1) the intervention and control arms, and (2) the intervention period and baseline.

**Results:**

With 2576 and 2536 pregnancies from 66 and 65 clusters in the intervention and control arms, respectively, the estimated risk ratios for receiving any ANC was 1.05 (95% CI 1.02 to 1.07), four or more ANC visits was 1.25 (95% CI 1.08 to 1.43) and ANC initiated in the first trimester was 1.11 (95% CI 1.02 to 1.19), relative to the controls; no differences in institutional delivery were found. However, both arms achieved large improvements in institutional delivery, compared with baseline. Monitoring data show that 19% and 2% of registered pregnancies received at least eight ANC contacts in the intervention and control arms, respectively. Six clusters, three from each arm had to be dropped in the last 2 years of the trial.

**Conclusions:**

Proactive home visits increased ANC and the number of antenatal contacts at the clinic and community levels. ANC and institutional delivery can be increased when provided without fees from professional CHWs in upgraded primary care clinics.

**Trial registration number:**

NCT02694055.

**WHAT IS ALREADY KNOWN ON THIS TOPIC**

- Available evidence suggests that a vast majority of pregnant women in the low-income and middle-income countries receive at least one antenatal care visit with a skilled provider. However, only about half receive at least four visits and even fewer achieve the current WHO recommendation of at least eight contacts either through outreach programmes or health worker involvement. Community health workers have been shown to improve access to quality services, including antenatal care and institutional delivery.

**WHAT THIS STUDY ADDS**

- Evidence from the trial demonstrates that home visits by a community health worker, when combined with user fee removal, professional provider and expanded primary care, together provide a feasible pathway to improve access to any antenatal care, four or more antenatal visits, and initiation of early antenatal care. Home visits could also help achieve eight or more antenatal contacts.

**HOW THIS STUDY MIGHT AFFECT RESEARCH, PRACTICE OR POLICY**

- The national health system of many countries, including Mali face challenges in achieving universal coverage of antenatal care and implementing the revised WHO recommendation. The evidence generated by this community-based trial is timely and will inform policies that can be implemented despite contextual challenges, in similar settings. Future research should focus on the need for further quality improvement of clinic-based care, and the potential value of implementing community-based intermittent preventive treatment of malaria using sulfadoxine-pyrimethamine.

## Introduction

Considerable variation in the coverage of antenatal care (ANC) exists between countries.^1^ In low-income and middle-income countries (LMICs), although 85% of pregnant women receive at least one ANC visit with a skilled provider, only 58% receive at least four visits.^2–4^ In Mali, these figures were 80% and 43%, respectively, in 2018.^5^ Reasons for poor coverage of ANC include financial barriers (formal and informal fees for service), geographical barriers and inadequate staff to provide high-quality, respectful care.^6 7^ ANC performed by a skilled provider helps in early identification of complications in pregnancy and delivery, provides nutritional supplementation, prophylactic therapy and counselling for healthy pregnancy.^8^ Effective antenatal counselling could also facilitate institutional delivery, which has been found to reduce the risk of adverse maternal and neonatal health outcomes, including mortality.^9 10^

In LMICs, facility-based ANC is low for a variety reasons.^11–13^ Community health workers (CHWs) have been shown to improve access to quality services, including ANC and institutional delivery.^14–17^ However, there is little agreement on the best package or delivery strategy of services to optimise impact. In 2018, the WHO guidelines on CHW programmes highlighted the research need to better understand CHW workflow that would optimise community engagement and care.^18^ The guidelines identified measuring the effect of home visits and in-home care by CHWs on access to care and mortality as a research priority. Thus, robust evidence from randomised studies on the effect of CHW home visits on access to pregnancy care, in the clinic as well as in the community, is needed to design effective CHW programmes.

The Proactive Community Case Management (ProCCM) cluster randomised controlled trial^19^ in Mali was designed to address whether proactive case detection home visits by CHWs, including the proactive detection of pregnancies, can improve ANC indicators and institutional delivery at a population level, compared with services delivered through a fixed health post when combined with a package of interventions designed to remove financial, geographical and clinical barriers to care. Given the nature of the intervention, an unblinded clustered randomised design was used because it was most pragmatic and feasible to implement, while retaining some of the advantages of a randomised design. A clustered design was necessary to prevent contamination between study arms, as it would not have been technically feasible to ensure that a CHW visiting one individual did not also encounter their neighbours in need of health service. In this manuscript, we present the secondary outcomes of the trial, using complementary sources of annual surveys and routine monitoring data. We additionally assessed the receipt of eight or more antenatal contacts as recommended by the WHO.^20^

## Methods

### Trial design and participants

The ProCCM trial was conducted in the health district of Bankass in central Mali, located about 600 km northeast of the national capital Bamako. The 2016 population of the study area was approximately 100 000, which composed of seven contiguous health catchment areas. Each area was served by one primary health centre (PHC) and a secondary referral hospital that was located within the district but outside the study area. Households in the study area generally included extended family ties and were geographically colocated within one family compound. Women were eligible to participate if they were between 15 and 49 years and permanent residents with no plans to relocate out of the study area during the trial. Once enrolled women remained in the trial even if they aged beyond 49 years.

Villages and hamlets within 1 km from one another were grouped into clusters. A total of 160 individual villages and hamlets were grouped into 137 unique clusters that covered the seven catchment areas entirely.^19^ Clusters were stratified by health catchment area and distance to the nearest PHC. Within each stratum, clusters were randomly assigned to the intervention or control arm using a computer-generated random number.^19^ Randomisation was conducted by a research team member in the USA who was blind to the identity and precise geographic location of clusters. During the trial, violence affected central Mali, including the study area. Consequently, six clusters, three from each arm had to be dropped in the second and third year of the trial. The manuscript presents information following the recommendations of the Consolidated Standards of Reporting Trials (CONSORT) statement extended to cluster randomised trials.^21^

### Description of the intervention

The intervention was implemented at the cluster level and described in detail in the protocol as well as in the online supplemental figure 1.^19^ Briefly, trained CHWs were deployed in the intervention clusters who conducted home visits for at least 2 hours each day, 6 days each week, with the goal of visiting each household at least twice every month to proactively find cases in the community. During the home visits, CHWs provided a comprehensive set of primary care services, including counselling, diagnostics, treatment and referral. They screened women of reproductive age for pregnancy, enquired the date of the last menstrual period (LMP) and offered home pregnancy testing to those more than 6 weeks beyond their LMP. Those who tested negative were offered preconception counselling, family planning counselling and/or contraceptive services in accordance with their choices. Those who tested positive, were enrolled by their CHW in a series of home-based and facility-based ANC visits and provided pregnancy-related education and counselling. CHWs visited pregnant women in their homes every 2 weeks until 8 months of gestation, thereafter weekly until delivery, during which they screened for danger signs and urgently arranged for facility-based care when necessary.

10.1136/bmjgh-2022-011071.supp2 Supplementary data

In the control clusters, CHWs provided the same package of primary care services as their counterparts in the intervention arm but only at their fixed community health sites, for at least 4 hours each day, 6 days each week. During the visits, pregnant women were reminded of their follow-up appointments and encouraged to return in a timely manner.

Across both arms, professionalised CHWs were trained, supervised, paid and deployed within each cluster, targeting one CHW for a population of approximately 700, in line with Mali’s national community health strategy.^22^ CHWs were expected to be promptly available on-call. Primary care clinic teams and infrastructure were redesigned and upgraded. A smartphone-based mobile application was developed to assist CHWs in both arms. It was designed to serve as a job aid to guide the CHWs through appropriate case management protocol, send task reminders and to track services rendered. All services were provided without fees. All residents in the study area, including visitors, were eligible to receive the healthcare, without fees.

### Data sources

The primary data source was the annual household surveys conducted at baseline (December 2016–January 2017), and at approximately 12 months (February–March 2018), 24 months (March–May 2019) and 36 months (February–April 2020) of the intervention period. The surveys included geographical coordinates at the entrance to a family compound, a household roster and modules on household characteristics including assets, which were administered to the female head of household or another household member at least 18 years of age. For some household characteristics such as floor and wall materials, etc, the interviewer also observed and made notes in the household roster. The surveys included a module for eligible women to collect information about care received during the most recent pregnancy, among other topics such as sociodemographics, contraceptive use, birth history, child health and service utilisation.^23^ For the baseline survey, women were asked about their most recent delivery, while for the subsequent ones, women were asked about deliveries in the year preceding the survey. All participating women in a cluster who delivered between surveys were included.

CHWs mobile application-based monitoring data, collected throughout the trial, was a second source of data for this analysis. The application was prepopulated using the baseline census data, including individual unique identifiers and demographic information, which allowed the CHWs to access the records in their service delivery zone. During each encounter with a patient, the CHW identified the individual or registered newborns, new patients, before selecting the appropriate form in the application for the specific health concern. Thereafter, the CHWs registered the services they provided. From the CHW mobile application data, we extracted the services provided to pregnant women and counted all ANC contacts to supplement information from the survey data. Pregnancies were counted if a CHW recorded it in the pregnancy diagnosis of the women or recorded it as a community-based or facility-based ANC visit. To limit the risk of inaccurate reporting and as part of their supervision protocol, dedicated CHW supervisors visited households in each CHW catchment area monthly and enquired patients about CHW services.

### Outcomes and covariates

The secondary outcomes reported in this analysis were obtained at the individual level and defined as: (A) any ANC received from a skilled provider (y/n); (B) four or more total ANC consultations with a skilled provider (y/n); (C) first trimester enrolment for ANC with a skilled provider (y/n); (D) took at least three doses of intermittent preventive treatment of malaria in pregnancy (IPTp3, y/n) and (E) institutional delivery (y/n). These outcomes were derived from self-reports to specific survey questions. In the context of the trial, a skilled provider was considered as a doctor, nurse, midwife, matron or a CHW and an institution was considered as a hospital (national, regional or referral), dispensary, maternity home, PHC, private hospital, private clinic or a treatment room. ‘Visits’ in this manuscript refer to visits to any of these institutions. In order to examine adherence to the revised WHO recommendations,^20^ we created eight or more total ANC (community or facility-based) contacts using the CHW application data. The annual surveys were not designed to effectively capture community-based ANC contacts.

As a measure of household socioeconomic status, we adapted the Demographic and Health Surveys method and created a wealth index based on livestock and durable goods ownership data collected in the annual household surveys, using principal component analysis.^24^ For each individual, we used information from the first survey wave in which her household enrolled in the trial. Household distance to the nearest PHC was determined using orthodromic (great-circle) distance estimates between family compounds and PHCs, using their respective GPS coordinates. Maternal age was calculated using self-reported dates of birth and survey. Marital status, education and occupation at the time of entry to the trial were used as covariates.

### Statistical analysis

This analysis included deliveries reported in the 24-month and 36-month surveys only, because for many deliveries reported in the 12-month survey, some period of the pregnancy likely occurred in the preintervention period. We compared the characteristics of participants included in the analysis by trial arms. We reported the proportion of each outcome, by trial arms and years. Using the CHW application data, we also examined four or more and eight or more ANC contacts for pregnancies between February 2018 and February 2020, to align with the period of the survey data used.

With individual deliveries as the unit, we performed intention-to-treat (ITT) analysis using multilevel logistic regression adjusting for the year of the trial. We used a random intercept to account for clustering of participants within a village cluster and quantified appropriate standard errors. Because the log-binomial models to generate risk ratio (RR) did not converge, we estimated OR from the logistic regression, which was converted to RR using the method proposed by Zhang and Yu.^25^ Because this approach may not generate appropriate estimates in the presence of large confounding,^26^ we additionally fit Poisson regressions to verify the RRs generated using the Zhang and Kai method. However, we do not report the Poisson RRs because Poisson-based errors overestimate the binomial errors when the outcome is common, as in this trial. We report the effect of intervention on each of the secondary outcomes comparing deliveries between the intervention and the control arms. As our interest was to examine the impact of the intervention on individual participants, participant-average treatment effect was quantified because it represents the population impact of switching from the control to the intervention clusters. We used recent methods recommended by Kahan *et al* to quantify the participant-average treatment effect.^27^

We examined heterogeneity of the intervention effects by strata of three prespecified factors: (A) distance to the nearest primary healthcare facility (5 km or less vs more than 5 km); (B) cluster population at baseline (less than 700 people vs 700 or more) and (C) household wealth (quintiles). Heterogeneity was measured using an interaction (product) term between intervention assignment and each of these prespecified factors.

In addition to the ITT analysis, we conducted a per-protocol analysis, using the inverse probability weights adjustment method, proposed by Hernán and Robins.^28^ Protocol adherence in the intervention arm was defined as any woman in the household reported having received two or more home visits by the CHW in the month prior to the survey. In assessing protocol adherence, we assumed that the pattern of CHW visits in the month prior to the survey was summarily reflective of the pattern throughout the year. We first estimated the probability of protocol adherence for all pregnancies in the intervention arm, using two logistic models: one with no covariates and another with all covariates that are listed in the covariate section. The ratio of the two probabilities generated the (stabilised) weights for the participants in the intervention arm, whereas participants in the control arm were assigned a weight of 1. We then fit the same models as in the ITT analysis, additionally including the stabilised weights.

We also compared outcomes reported in the 24-month and 36-month surveys with that of the baseline. As in the ITT analysis, to allow adequate time for the intervention to have an effect we excluded deliveries reported in the 12-month survey.

Finally, we performed sensitivity analyses by further adjusting the main models with additional covariates where some differences were observed by trial arms. In a second sensitivity analysis, we included deliveries reported in the 12-month survey to the main analytical cohort, to examine potential selection bias due to their exclusion. Separately, we compared the key characteristics of the participants who were excluded (reported delivery in the 12-month survey) with those who were included (reported delivery in the 24-month and 36-month surveys). We also observed that some control households reported having occasional CHW home visit(s) in non-adherence to the protocol. Thus, we conducted a third sensitivity analysis by excluding the control participants who reported CHW home visits in the month preceding the surveys. A post-hoc power calculation accounting for the design effect due to clustering is presented in online supplemental table 1 with accompanying description of the methods. All hypothesis tests were two tailed at the 5% significance level. Analyses were conducted using Stata V.17.0 (StataCorp).

10.1136/bmjgh-2022-011071.supp1 Supplementary data

### Role of the funding sources

The funders of the study had no role in study design, data collection, data analysis, data interpretation or writing of the report. The corresponding author had full access to all the data in the study and had final responsibility for the decision to submit for publication.

### Patient and public involvement

The study was designed and implemented in partnership with national, district and local health officials of the Malian Ministry of Health. Bankass health district was chosen in consultation with the Ministry of Health for three reasons: (1) healthcare utilisation (prenatal and curative consultations) was low and under-5 mortality was high; (2) there were no overlapping interventions by other non-governmental organisations at the time or intended for the period of the trial and (3) local authorities were highly engaged and interested in collaborating on study implementation. Research questions and outcome measures were also chosen in consultation, to answer questions of key concern to government partners for informing the design of the national strategic plan for iCCM scale-up, including whether the intervention is equitable, cost-effective and affordable at scale. Community consultation and permission were sought prior to trial commencement in meetings with representatives of the village clusters, such as village chiefs and their advisories, politico-administrative authorities, religious leaders and representatives of women’s and youth associations. Representatives then communicated with community members via open public meetings. Findings will be disseminated via workshops at all levels of local, regional and national representation.

## Results

**Figure 1:**
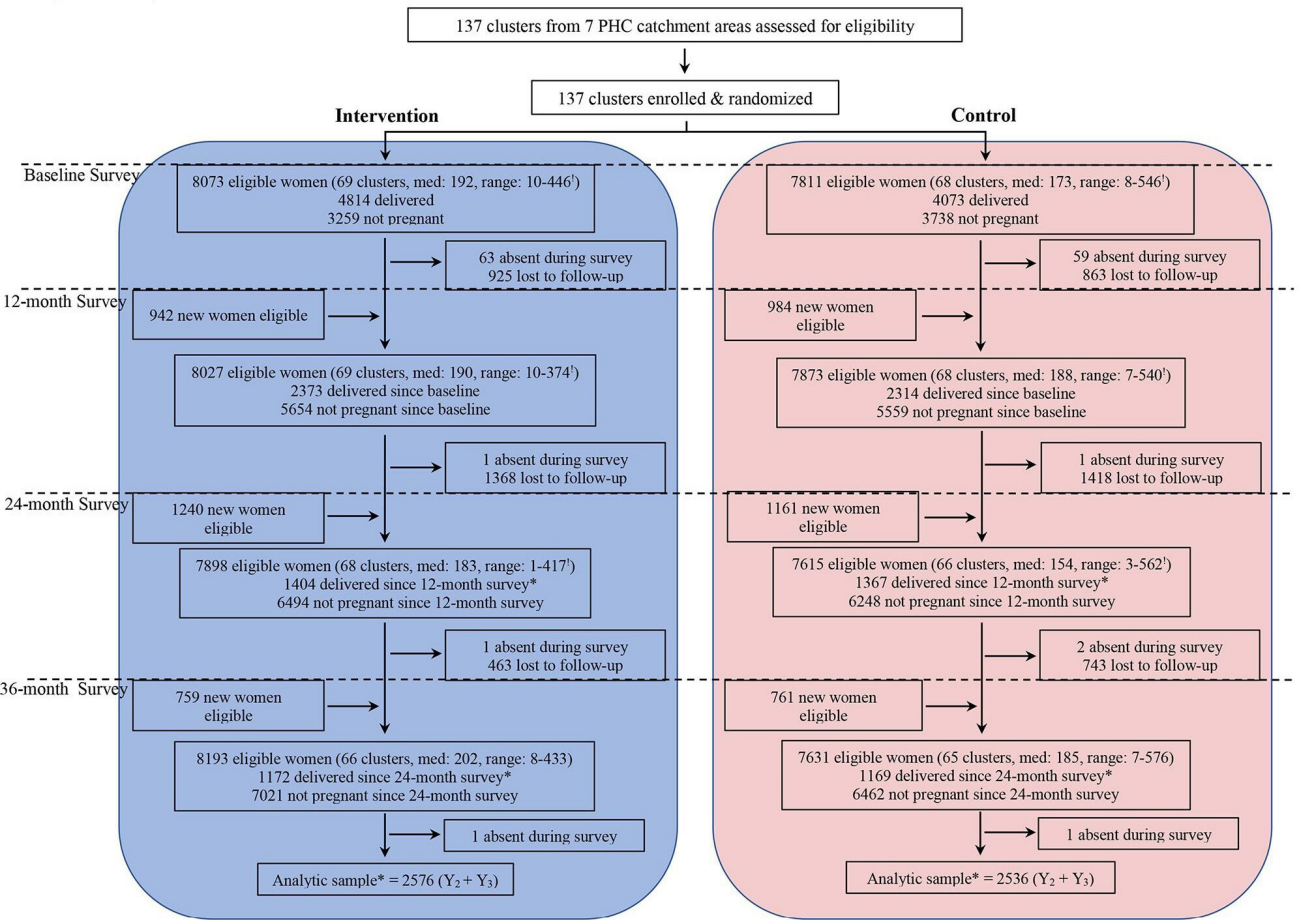
Trial profile. Women were eligible if they were between 15 and 49 years and permanent residents with no plans to relocate out of the study area during the trial. !Med refers to the median cluster size in that arm in that year; range is the minimum and the maximum number of participants in the smallest cluster and the largest cluster, respectively. *Analytical sample for each arm is the sum of the eligible women who delivered since the 12-month and the 24-month survey, respectively. PHC, primary health centre.

The CONSORT diagram presents the number of clusters as well as the number of women who were eligible, completed the household surveys, lost to follow-up and included in the analysis, for each year and by trial arm (figure 1). The trial began with 69 and 68 clusters in the intervention and control arms, respectively. However, 2576 and 2536 pregnancies from 66 and 65 clusters in the intervention and control arms, respectively, were included in the analysis for the comparison between arms. Three clusters had to be dropped from each arm, as violence affected the study area. Table 1 presents characteristics of participants at the time of entry to the trial.

**Table 1:** Individual women, household and cluster level characteristics, by trial arms and overall

Characteristics	Intervention		Control		Total	
	n	%*	n	%*	n	%
Total participants†	2491	50.5	2441	49.5	4932	100
Age at entry (years)						
15–19	297	11.9	325	13.3	622	12.6
20–34	1772	71.11	1753	71.8	3525	71.5
35–49	365	14.7	320	13.1	685	13.9
Missing	57	2.3	43	1.8	100	2.0
Marital status						
Married: monogamous	1355	54.4	1276	52.3	2631	53.4
Married: polygamous	1079	43.3	1105	45.4	2184	44.3
Never married/widowed/divorced/separated	57	2.3	60	2.5	117	2.4
Ethnicity						
Dogon	2169	87.1	2088	85.5	4257	86.3
Peulh	18	0.7	23	0.9	41	0.8
Others	19	0.8	49	2.0	68	1.4
Missing	285	11.4	281	11.5	566	11.5
Education						
Any school (Madrasah or French)	261	10.5	291	10.0	552	11.2
No formal education	2072	83.2	2010	82.3	4082	82.8
Missing	158	6.4	140	5.7	298	6.1
Respondent’s occupation						
Housewife	2041	81.9	1987	81.4	4028	81.7
Small business or trader	441	17.7	441	18.1	882	16.0
Other	0	0	4	0.2	4	0.1
Missing	9	0.4	9	0.4	18	0.4
Household wealth quintile						
Poorest	555	22.3	483	19.8	1038	21.1
Poor	473	19.0	476	19.5	949	19.2
Middle	473	19.0	477	19.5	950	19.3
Rich	484	19.4	492	20.2	976	19.8
Richest	499	20.0	504	20.7	1003	20.3
Missing	7	0.3	9	0.4	16	0.3
Residential distance to nearest primary health centre (kilometres)						
≤5	1026	41.2	1195	49.0	2221	45.0
>5	1465	58.8	1246	51.0	2711	55.0
Cluster population at baseline						
<700	756	30.4	927	38.0	1683	34.1
≥700	1735	69.7	1514	62.0	3249	65.9

*There were 4932 women in trial years 2 and 3 who had 5112 pregnancies. 180 women reported consecutive pregnancies.

†Percentage for each characteristic is out of the total in that arm, except for the very first row.

**Figure 2:**
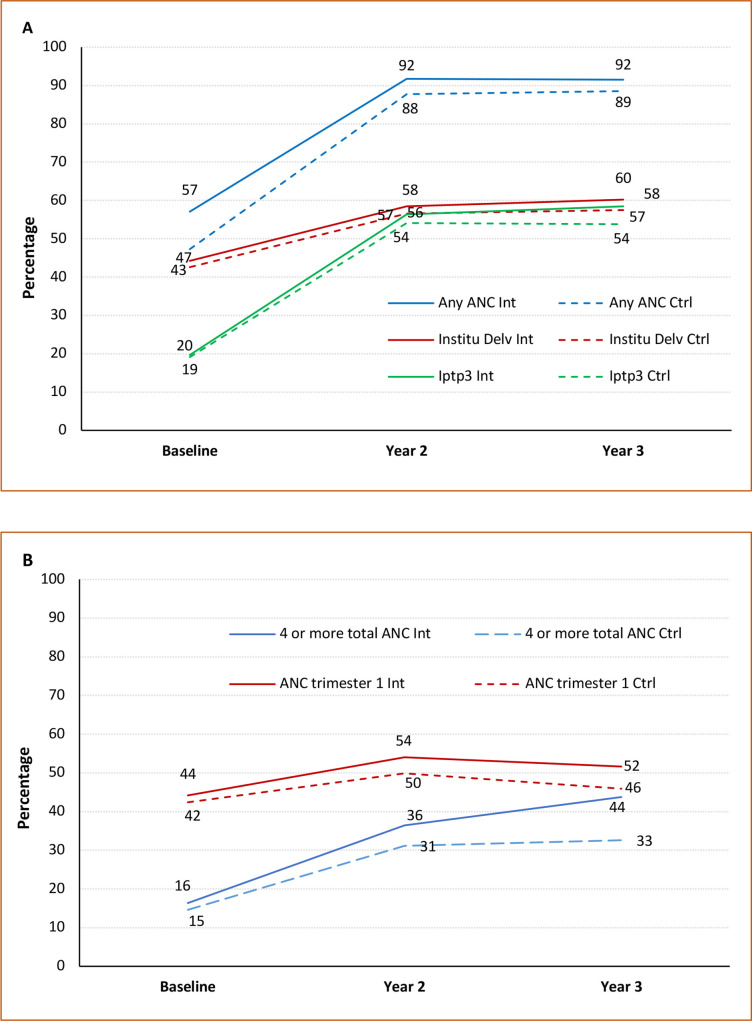
Distribution of the maternal health indicators by trial arms. (A) Any antenatal care (ANC), institutional delivery, preventive treatment for malaria; (B) four or more antenatal visits and ANC initiated in the first trimester.

Across both arms, relatively large increases were observed for all pregnancy-related outcomes reported in the 24-month and 36-month surveys compared with baseline (figures 2A, 2B). In the intervention arm, the percentage of women who received any ANC from a skilled provider, IPTp3 or had institutional delivery was 1–4 points higher than women in the control arm (figure 2A). Relatively larger differences, of 6–11 points, between arms were observed for the percentages of women who received four or more ANC and those who initiated ANC in the first trimester (figure 2B). Numerical estimates for each outcome along with the 95% CIs are presented in the online supplemental table 2. CHW mobile application data show that in the corresponding period, 2778 registered pregnancies received any ANC contact. Of these, 45% received four or more ANC contacts in the intervention arm, compared with 7% in the control arm; 19% received eight or more ANC contacts in the intervention arm, compared with 2% in the control arm (online supplemental table 3).

A comparison between arms, using ITT analysis shows the RR for any ANC was 1.05 (95% CI 1.02 to 1.07), for four or more total ANC visits it was 1.25 (95% CI 1.08 to 1.43) and for ANC initiated in the first trimester it was 1.11 (95% CI 1.02 to 1.19), relative to the controls (table 2). For IPTp3 and institutional delivery, while the point estimates suggest a small positive effect of the intervention, the CIs did not exclude 1. Protocol adherence was 43.2% and the per-protocol analysis yielded similar results (table 2). In addition to the relative differences, the difference between arms shown in figures 2A, 2B reflect the absolute differences because of little effect of confounding as demonstrated by the sensitivity analysis below and also because the outcomes are common. There was no evidence of heterogeneity of the observed effects by distance to the nearest primary healthcare facility, cluster population at baseline or household wealth at entry to the trial (online supplemental table 4).

**Table 2:** Comparison of the intervention with the control arm and estimation of the intervention effect on indicators of antenatal care and institutional delivery

Outcomes	Intervention	Control	Intention to treat*	Per protocol*
	n_1_/N_1_† (%)	n_2_/N_2_† (%)	RR (95% CI)‡	RR (95% CI)‡
Received any antenatal care	2361/2576 (91.7)	2235/2536 (88.1)	1.05 (1.02 to 1.07)	1.04 (1.01 to 1.07)
Four or more total antenatal visits	938/2360 (39.8)	711/2233 (31.8)	1.25 (1.08 to 1.43)	1.25 (1.08 to 1.44)
Antenatal care initiated in the first trimester	1216/2296 (52.9)	1053/2191 (48.1)	1.11 (1.02 to 1.19)	1.11 (1.03 to 1.20)
Intermittent preventive treatment of pregnancy	1397/2438 (57.3)	1305/2419 (53.9)	1.06 (0.97 to 1.15)	1.07 (0.97 to 1.15)
Institutional delivery	1506/2542 (59.2)	1421/2494 (56.9)	1.06 (0.91 to 1.20)	1.06 (0.91 to 1.20)

*Includes trial years 2 and 3.

†Counts with the outcome (n) out of the total participants (N) in the arm.

‡Each outcome was analysed separately and each model included a fixed effect for year of intervention, residential distance to the nearest primary health centre, baseline population of the cluster and a random intercept for cluster. The CI accounted for clustering of observations.

RR, risk ratio.

A comparison of outcomes reported in the 24-month and 36-month surveys with the baseline is presented in table 3. Relative to baseline, the estimated RRs for the last 2 years of the trial, was 1.83 (95% CI 1.78 to 1.86) for any ANC, 2.59 (95% CI 2.28 to 2.91) for four or more total ANC visits, 1.15 (95% CI 1.06 to 1.25) for ANC initiated in the first trimester, 3.42 (95% CI 2.98 to 3.84) for IPTp3 and 1.54 (95% CI 1.41 to 1.66) for institutional delivery (table 3).

**Table 3:** Comparison of the intervention period with the baseline and estimation of the effect of intervention period on indicators of antenatal care and institutional delivery

Outcomes	Trial years 2 and 3 vs baseline		
	Trial years 2 and 3, n_1_/N_1_* (%)	Baseline, n_2_/N_2_* (%)	RR (95% CI)†
Received any antenatal care	4596/5112 (89.9)	4674/8887 (52.6)	1.83 (1.78 to 1.86)
Four or more total antenatal visits	1649/4593 (35.9)	533/3420 (15.6)	2.59 (2.28 to 2.91)
Antenatal care initiated in the first trimester	2269/4487 (50.6)	1468/3383 (43.4)	1.15 (1.06 to 1.25)
Intermittent preventive treatment of pregnancy	2702/4857 (55.6)	1444/7437 (19.4)	3.42 (2.98 to 3.84)
Institutional delivery	2927/5036 (58.1)	2901/6668 (43.5)	1.54 (1.41 to 1.66)

*Counts and proportions with the outcome, out of the total participants in the two comparison groups.

†Each outcome was analysed separately and each model included a fixed effect for year of intervention, residential distance to the nearest primary health centre, baseline population of the cluster and a random intercept for cluster. The CIs accounted for clustering of observations.

RR, risk ratio.

### Sensitivity analysis

In sensitivity analysis, these results remained unchanged to additional adjustment for covariates (online supplemental table 5). Further, results show that there is minimal influence of exclusion of women who reported a delivery in the 12-month survey (online supplemental table 6). In online supplemental table 7, we have shown the distribution of key characteristics of the participants who reported a delivery in the 12-month survey and those who reported deliveries in the 24-month or 36-month surveys. About 13% and 12% of the participants in the control arm reported that CHWs visited their homes, in the 24-month and 36-month surveys, respectively. To eliminate potential effects of contamination of the results, we fit the same models after excluding control participants who had CHW home visits, and the estimated RRs for intervention were largely the same (online supplemental table 8). The participant-average treatment effects are shown in online supplemental table 9. For the intervention versus control comparison, the participant-average treatment effects are virtually identical to those based on the ITT analysis presented in table 2. However, the participant-average treatment effects for the comparison of the outcomes reported in the 24-month and 36-month surveys with that of the baseline shown in the online supplemental table 9 are slightly attenuated when compared with the results shown in table 3.

## Discussion

The findings indicate that proactive home visits had a positive effect on pregnant women receiving any ANC, four or more ANC visits and initiation of ANC in the first trimester, when compared with service delivery only through fixed community health sites. The impact on receiving four or more ANC visits and initiation of care early in pregnancy appears to be larger than the impact on receiving any care during pregnancy. Additionally, the CHW mobile application data shows a higher proportion of women in the intervention arm who received any ANC received eight or more ANC contacts, compared with women in the control arm. Three potential mechanisms may have contributed towards improved ANC with home visits. First, proactive case detection may have identified more pregnancies, and sooner, thereby initiating ANC early and providing more opportunity throughout the pregnancy to achieve four or the WHO recommended eight antenatal contacts. Second, home visits may have provided direct opportunities to conduct community-based antenatal visits, removing geographic barriers and opportunity costs to care (eg, time lost from work) further by bringing community-based services into the home. Third, home visits may have increased antenatal clinic attendance by building trust, counselling and encouraging appointment attendance, as well as by supporting patients to overcome any barriers to facility-level care.

Proactive home visits do not appear to have a significant effect on preventive treatment for malaria during pregnancy or institutional delivery. The elevated RR (1.06) for the effect of intervention on institutional delivery and the 95% CI (0.91 to 1.20) could be interpreted as a small magnitude of impact of the intervention,^29^ which the trial did not have the sample size to estimate with higher precision. The results may suggest the need for further quality improvement of clinic-based care, and point to the potential value of integrating malaria prophylaxis into community-based ANC visits. Recall bias could also be an explanation for the IPTp3 results. Pregnant women could have misreported or forgotten during the surveys exactly how many doses of a specific medicine she took over approximately 9 months. This is, however, less likely to happen for the other outcomes like the number of times she went for check-up during pregnancy or where she delivered her last child, as these are relatively significant events and easier to recall.

A comparison of outcomes reported in the 24-month and 36-month surveys with those reported at the baseline shows consistent trend of improvement across all outcomes. For example, the likelihood of receiving four or more ANC visits and IPTp3 was 2.6 times and 3.4 times more during the intervention period than at baseline, respectively. In comparison, the magnitudes of the differences between arms were relatively small. The relatively small incremental effect of proactive home visits by CHWs is important because it is over and above the considerable changes that simultaneously occurred across both arms. The comparatively large change since baseline could likely be driven by the package of interventions provided across arms, including removing point of care fees, supporting paid, professional CHWs to provide the same package of care in every village, and upgrading primary care clinics and teams. Each of these evidence-based strategies implemented in both study arms were designed to remove key barriers, and to improve access to early and complete ANC.^30^ The additional component of proactive home visits in the intervention arm potentially further enhanced access to care during pregnancy.

In the Malian context, universal coverage of ANC in general, including the implementation of the WHO recommendation of eight ANC, is plagued by financial, geographical, infrastructural, gender-based and human resources barriers to care. The Government of Mali has integrated the revised WHO recommendation of eight contacts in its maternal health policies, which was validated by the Malian Ministry of Health in 2019, although its implementation is yet to begin. As the national health system faces challenges in achieving the revised WHO recommendation, the country is currently seeking evidence-based strategies to reach these maternal healthcare goals. Furthermore, the Malian Government in 2022 recognised CHWs as professionals within the national healthcare system, and detailed a regulatory framework for supporting them. In this context, the government is in the process of scaling up community health services nationally, with a particular focus on improving maternal and child health outcomes. The evidence generated by this community-based trial will prove timely to inform these national efforts. The findings could also be informative (ie, generalisable) to other governments, in comparable settings faced with contextual challenges, seeking to optimise their community healthcare systems in alignment with recent WHO recommendations to improve access to ANC.

The trial had some limitations, key among which are a lack of information on maternal complications and mortality. Hence, we cannot assess the effect of improvement in ANC on maternal morbidity and mortality, which would require a much larger study population and was beyond the scope of this trial. Second, this analysis used two data sources because the survey data captured almost none of the community-based ANC visits, while the CHW mobile application data recorded the community-based ANC visits, but did not effectively capture the clinic-based ANC. These two sources, each with their own limitations, together provided information on ANC contacts. Third, inaccurate recall of the number of ANC visits or timing of the first ANC is a possibility. If there is recall error and it is differential by arm, the observed results could be biased. If the recall error is non-differential by arm, the results are more likely to inflate the standard errors and affect precision.^31^ Given that participant characteristics in the two arms were relatively similar, there is little reason to support differential recall error by arm. Fourth, during the trial period, violence affected central Mali, including the study area. Consequently, six clusters, three from each arm had to be dropped in the second and third year of the trial. The trial protocol was adapted to cater to the volatile environment, including deploying mobile clinics to clusters cut-off from their nearest health centre, and deploying mobile CHWs to clusters where the CHWs had to vacate. Clusters that were dropped were similar to those that were retained, in their baseline characteristics. Thus, any one arm is unlikely to be differentially affected by the loss of participants from these clusters.

This trial has several strengths, including the randomised design that likely increases internal validity of the findings. Availability of several indicators of pregnancy care in a longitudinal cohort allowed investigation of the effect of the intervention beyond the primary outcome. To strengthen the inferences, we performed several sensitivity analyses to explore the possibility of alternative explanations for the observed effect of the intervention. Clusters were randomly assigned to the trial arms while outcomes were assessed on individual women. Thus, there may be potential for confounding due to the different levels of randomisation and outcome measurement, giving rise to imbalanced arms that could be more than by chance. To examine imbalance, we compared the arms by key characteristics of the participants, households and clusters in the analytical cohort. The arms were largely homogeneous. Additionally, we adjusted the main results with the key characteristics shown in table 1; the results remained unchanged. As participants that reported a pregnancy in the 12-month survey were excluded from the main analytical cohort, we explored if selection bias could explain the observed results. A comparison of key characteristics among those excluded (12-month survey participants) with those who were included (24-month and 36-month survey participants) does not show considerable difference. Including 12-month survey participants to the main analytical cohort did not alter the effect of intervention. Furthermore, a proportion (12%–13%) of the control participants received proactive home visits by CHWs. We investigated whether information bias (ie, those in the control group who were exposed to the intervention) affected the results. Excluding participants in the control arm who received proactive home visits did not change the results. While we cannot completely rule out biases, the longitudinal design and randomised allocation of intervention minimises that potential and multiple sensitivity analyses demonstrated robustness of the findings. This gives reasonable confidence that the observed results are more likely to be true effects.

The ProCCM trial found that home visits improved access to any ANC, four or more ANC visits, initiation of early pregnancy care, as well as eight or more ANC contacts as measured using monitoring data. Compared with baseline, large improvements in ANC, malaria prophylaxis in pregnancy and institutional delivery, were achieved during the trial years when patients received care without user fees, from a professional CHW and with upgraded primary care clinics. The evidence from this trial is significant for public health practitioners and policy makers because it demonstrates a model that is feasible to implement, even in difficult contexts and in the setting of violent conflict. Many countries continue to struggle to achieve WHO targets for ANC. Home visits, when combined with user fee removal, professional CHWs and expanded primary care, together provide a promising pathway for progress.

## Data Availability

Data are available on reasonable request. Household survey panel data that is deidentified will be made available to external researchers upon reasonable request to Muso, by contacting Ari Johnson ajohnson@musohealth.org; Ari.Johnson@ucsf.edu.
