# Supplementary material for: Effect of community health worker home visits on antenatal care and institutional delivery: an analysis of secondary outcomes from a cluster randomised trial in Mali
Source: BMJ Glob Health. 2023 Mar 22;8(3):e011071. doi: 10.1136/bmjgh-2022-011071 (PMC10040070; doi:10.1136/bmjgh-2022-011071)
Supplement: Supplementary data [file bmjgh-2022-011071supp002.pdf]

Supplementary Figure 1. A description of the intervention in the Proactive Community Case Management Trial.

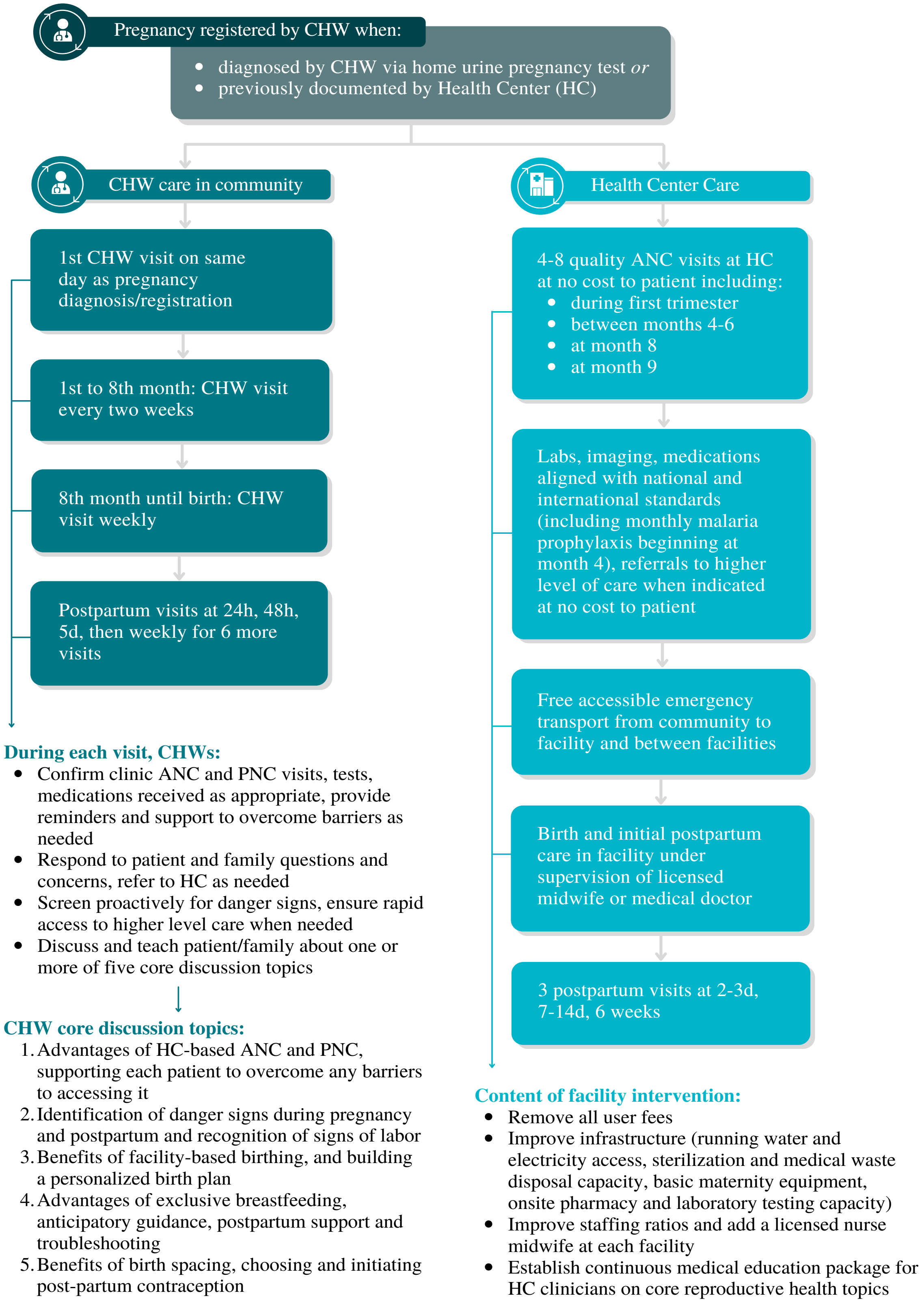

Note: Community Health Workers in intervention clusters proactively visited all homes to provide care. In control clusters, Community Health Workers provided the same services at their fixed community health post to care-seeking patients. The Health Center-level intervention was the same for both clusters.
