# Supplementary material for: Effect of community health worker home visits on antenatal care and institutional delivery: an analysis of secondary outcomes from a cluster randomised trial in Mali
Source: BMJ Glob Health. 2023 Mar 22;8(3):e011071. doi: 10.1136/bmjgh-2022-011071 (PMC10040070; doi:10.1136/bmjgh-2022-011071)
Supplement: Supplementary data [file bmjgh-2022-011071supp001.pdf]

Supplementary information

Sample size

Post-hoc power calculation for the secondary outcomes of the trial is presented in supplementary table 1. To estimate power, we used the actual sample size specific to the intervention-outcome pair that was included in the analysis along with the intracluster correlation of that outcome in that sample, as shown in the supplementary table 1. The trial included a total of 137 clusters and assuming approximately equal cluster size, we estimated the design effect as shown in Equation 1, where m refers to the average cluster size and ρ is the intracluster correlation. The effective sample size was obtained by dividing the actual sample size with the design effect.

$$Design\ effect = 1 + (m-1)\rho \ldots\ldots\ldots\ldots\ldots\ldots Eq\ 1.$$

We estimated the power using the effective sample sizes, observed proportions of the outcomes and the risks for each intervention-outcome pair using the software PASS 2020 [1]. We used 1:1 ratio of participants in the two arms and accounted for two-level data structure with randomization at the cluster (highest) level. A priori power calculation, for a given sample size, gives the probability of detecting a pre-specified effect size at the 95% confidence level. However, this was a post-hoc power calculation after obtaining the results. In some instances, this study did not meet the minimum acceptable level of 80% power to detect the observed effect significantly.

Reference

1. NCSS LLC., *Power Analysis and Sample Size Software*. 2020: Kaysville, Utah, USA.

Supplementary Table 1. **Post-hoc power estimation for the outcomes.**

	Actual sample size	Intracluster correlation	Design effect	Effective sample size	Relative risk	Power (%)
*Intervention vs. control**						
Received any antenatal care	5112	0·1376	6·00	852	1·05	45·4
Four or more total antenatal visits	4,593	0·0867	3·82	1203	1·25	75·4
Antenatal care initiated in the 1st trimester	4,487	0·0276	1·88	2391	1·11	63·6
Intermittent preventive treatment of pregnancy	4857	0·0532	2·83	1715	1·06	21·9
Institutional delivery	5036	0·2597	10·29	490	1·06	11·6
Study years 2 and 3 vs· baseline						
Received any antenatal care	13999	0·2083	22·08	634	1·83	99·9
Four or more total antenatal visits	8013	0·0553	4·18	1917	2·59	99·9
Antenatal care initiated in the 1st trimester	7870	0·0168	1·95	4040	1·15	99·5
Intermittent preventive treatment of pregnancy	12294	0·0639	6·67	1842	3·42	99·9
Institutional delivery	11704	0·2185	19·45	602	1·54	99·9

* Assumption – 137 clusters with approximately equal size.

Kayentao K, *et al. BMJ Global Health* 2023; 8:e011071. doi: 10.1136/bmjgh-2022-011071

Supplementary Table 2. Distribution of the maternal health indicators and their 95% confidence intervals presented by trial arms as reported in the Figure 2 of the main document.

	Intervention	Control
**Outcomes**	**Proportion (95% CI)**	**Proportion (95% CI)**
Received any antenatal care		
Baseline	57.1 (55.7, 58.5)	47.3 (45.8, 48.8)
Year 2	91.7 (90.3, 93.2)	87.8 (86.0, 89.5)
Year 3	91.6 (90.0, 93.1)	88.5 (86.7, 90.4)
Four or more total antenatal visits		
Baseline	16.4 (14.7, 18.0)	14.6 (12.8, 16.4)
Year 2	36.4 (33.8, 39.0)	31.2 (28.9, 33.8)
Year 3	43.8 (40.8, 46.7)	32.6 (29.7, 35.5)
Antenatal care initiated in the 1st trimester		
Baseline	44.2 (41.9, 46.4)	42.4 (40.0, 44.9)
Year 2	54.0 (51.3, 56.8)	50.0 (47.0, 52.7)
Year 3	51.7 (48.6, 54.7)	46.0 (42.9, 49.0)
Intermittent preventive treatment of pregnancy		
Baseline	19.7 (18.4, 20.9)	19.1 (17.8, 20.4)
Year 2	56.4 (53.7, 59.0)	54.1 (51.4, 56.8)
Year 3	58.4 (55.5, 61.4)	53.8 (50.8, 56.7)
Institutional delivery		
Baseline	44.2 (42.6, 45.8)	42.6 (40.8, 44.4)

Year 2	58.4 (55.8, 61.0)	56.5 (53.9, 59.2)
Year 3	60.2 (57.4, 63.0)	57.5 (54.6, 60.3)

Supplementary Table 3. Proportion of pregnancies who received of four or more and eight or more antenatal contacts obtained from the Community Health Workers mobile application data, presented by trial arms.

			Trial years 2 and 3*					
			Intervention		Control		Total	
**Data source**	**Description**	**Indicator**	**n**	**%**	**n**	**%**	**n**	**%**
CHW application	Total (facility + community) contacts	Pregnancies that received antenatal contact	1725		1053		2778	
		4 or more contacts	778	45·1	78	7·4	856	30·8
		8 or more contacts	329	19·1	24	2·3	353	12·7
	Only community-based contacts	Pregnancies that received antenatal contact	1720		1045		2765	
		4 or more contacts	762	44·3	77	7·4	839	30·3
		8 or more contacts	319	18·6	24	2·3	343	12.4

* ANC contacts between February 2018 and February 2020 i.e., trial years 2 and three.

Kayentao K, *et al. BMJ Global Health* 2023; 8:e011071. doi: 10.1136/bmjgh-2022-011071

Supplementary Table 4. Heterogeneity of the effect of intervention on indicators of antenatal care and institutional delivery by distance to the nearest primary health care facility, cluster population at baseline, or household wealth.

	Received any antenatal care*	Four or more total antenatal visits*	Antenatal care initiated in the 1st trimester*	Intermittent preventive treatment of pregnancy*	Institutional delivery*
Residential distance to the nearest primary health care facility					
Interaction coefficient (95% CI)[ǂ]	0·23 (-0·42, 0·87)	-0·04 (-0·50, 0·43)	0·23 (-0·12, 0·58)	0·06 (-0·34, 0·46)	0·28 (-0·46, 1·01)
p-value	0·49	0·87	0·19	0·77	0·46
Cluster population at baseline					
Interaction coefficient (95% CI)	-0·05 (-0·72, 0·61)	0·22 (-0·24, 0·68)	-0·11 (-0·43, 0·21)	-0·03 (-0·42, 0·37)	-0·22 (-0·91, 0·47)
p-value	0·87	0·35	0·50	0·90	0·53
Household wealth quintiles					
Poor: Interaction coefficient (95% CI)	-0·14 (-0·71, 0·43)	0·02 (-0·38, 0·43)	0·29 (-0·06, 0·64)	-0·17 (-0·59, 0·25)	0·11 (-0·32, 0·55)
p-value	0·64	0·90	0·11	0·42	0·60
Middle: Interaction coefficient (95% CI)	-0·29 (-0·93, 0·34)	0·15 (-0·24, 0·54)	0·41 (-0·03, 0·85)	-0·26 (-0·64, 0·13)	-0·17 (-0·66, 0·32)
p-value	0·37	0·44	0·07	0·19	0·50
Rich: Interaction coefficient (95% CI)	-0·73 (-1·37, -0·09)	0·06 (-0·31, 0·44)	0·29 (-0·05, 0·63)	-0·17 (-0·54, 0·20)	0·16 (-0·30, 0·61)
p-value	0·03	0·74	0·10	0·36	0·50
Richest: Interaction coefficient (95% CI)	0·08 (-0·47, 0·64)	-0·11 (-0·49, 0·26)	0·52 (0·12, 0·93)	-0·38 (-0·74, -0·02)	0·14 (-0·31, 0·59)
p-value	0·77	0·55	0·01	0·04	0·54

* Each outcome was analyzed separately and each model included a fixed effects for intervention/control, year of intervention, a fixed effect for the interaction variable, a product term between intervention/control and the interaction variable, and a random intercept for cluster· The confidence intervals accounted for clustering of observations.

[ǂ] The coefficient and the p-value presented in the table are for the product term only.

Supplementary Table 5. Sensitivity analysis by additional adjustment of the main results in tables 2 and 3 with variables that showed some evidence of imbalance by study arms.

	RR (95% CI) *	
**Outcomes**	**Intervention/Control**	**Study years/Baseline**
Received any antenatal care	1·04 (1·01, 1·07)	1·75 (1·67, 1·80)
Four or more total antenatal visits	1·25 (1·08, 1·43)	2·44 (2·10, 2·80)
Antenatal care initiated in the 1st trimester	1·11 (1·03, 1·20)	1·15 (1·03, 1·28)
Intermittent preventive treatment of pregnancy	1·05 (0·96, 1·14)	2·99 (2·59, 3·40)
Institutional delivery	1·07 (0·92, 1·21)	1·49 (1·34, 1·62)

* The models included a fixed effect for year of intervention, residential distance to the nearest primary health center and baseline population of the cluster, maternal age, marital status and household wealth quintiles and random intercepts for clusters.

Supplementary Table 6. Sensitivity analysis by including year 1 participants to the analytical cohort.

	RR (95% CI) *	
**Outcomes**	**Intervention/Control**	**Study years/Baseline**
Received any antenatal care	1·04 (1·01, 1·07)	1·43 (1·40, 1·44)
Four or more total antenatal visits	1·22 (1·06, 1·38)	2·21 (2·01, 2·41)
Antenatal care initiated in the 1st trimester	1·12 (1·04, 1·19)	1·15 (1·05, 1·25)
Intermittent preventive treatment of pregnancy	1·08 (0·99, 1·11)	2·15 (2·01, 2·27)
Institutional delivery	1·06 (0·93, 1·18)	1·42 (1·32, 1·51)

* The models included a fixed effect for year of intervention, residential distance to the nearest primary health center and baseline population of the cluster.

Supplementary Table 7. Individual, household and cluster level characteristics, by trial arms and overall.

	Years 2 and 3				Year 1			
	Intervention		Control		Intervention		Control	
**Characteristics**	n	%*	n	%*	n	%*	n	%*
Total participants[#]	2491	50·5	2441	49·5	2373	50·6	2314	49·4
Age at entry (years)								
15 - 19	297	11·9	325	13·3	181	7·6	209	9·0
20 - 34	1772	71·11	1753	71·8	1556	65·5	1560	67·4
35 - 49	365	14·7	320	13·1	588	24·8	509	22·0
Missing	57	2·3	43	1·8	48	2·0	36	1·6
Marital status								
Married: monogamous	1355	54·4	1276	52·3	1246	52·5	1264	54·6
Married: polygamous	1079	43·3	1105	45·4	1058	44·6	1004	43·4
Never married/Widowed/Divorced/Separated	57	2·3	60	2·5	69	2·9	46	2·0
Ethnicity								
Dogon	2169	87·1	2088	85·5	5866	24·7	528	22·8
Peulh	18	0·7	23	0·9	4	0·2	7	0·3
Others	19	0·8	49	2·0	4	0·2	7	0·3
Missing	285	11·4	281	11·5	xx	xx	xx	76·6
Education								
Any school (Madrasah or French)	261	10·5	291	10·0	77	3·2	101	4·4
No formal education	2072	83·2	2010	82·3	709	30·0	678	29·3
Missing	158	6·4	140	5·7	xx	xx	xx	66·3
Respondent's occupation								
Housewife	2041	81·9	1987	81·4	1882	79·3	1875	81·0
Small business or trader	441	17·7	441	18·1	447	18·8	400	17·3
Other	0	0	4	0·2	26	1·1	33	1·4

Kayentao K, *et al. BMJ Global Health* 2023; 8:e011071. doi: 10.1136/bmjgh-2022-011071

Missing	9	0·4	9	0·4	18	0·8	6	0·3
Household wealth quintile								
Poorest	555	22·3	483	19·8	406	17·1	395	17·1
Poor	473	19·0	476	19·5	415	17·5	473	20·4
Middle	473	19·0	477	19·5	491	20·7	469	20·3
Rich	484	19·4	492	20·2	499	21·0	500	21·6
Richest	499	20·0	504	20·7	544	22·9	465	20·1
Missing	7	0·3	9	0·4	18	0·8	12	0·5
Residential distance to nearest primary health center (kilometers)								
≤ 5	1026	41·2	1195	49·0	1012	42·7	1138	49·2
> 5	1465	58·8	1246	51·0	1361	57·4	1176	50·8
Cluster population at baseline								
< 700	756	30·4	927	38·0	680	28·7	890	38·5
≥ 700	1735	69·7	1514	62·0	1693	71·3	1424	61·5

[‡] Percentage for each characteristic is out of the total in that arm, except for the very first row.

Supplementary Table 8. Sensitivity analysis by excluding control participants who received home visits from the analytical cohort.

	RR (95% CI) *	
**Outcomes**	**Intervention/Control**	**Study years/Baseline**
Received any antenatal care	1·05 (1·01, 1·07)	1·83 (1·78, 1.86)
Four or more total antenatal visits	1·25 (1·08, 1·43)	2·63 (2·32, 2·96)
Antenatal care initiated in the 1st trimester	1·10 (1·01, 1·19)	1·15 (1·05, 1·25)
Intermittent preventive treatment of pregnancy	1·07 (0·98, 1·16)	3·39 (2·94, 3·82)
Institutional delivery	1·06 (0·91, 1·21)	1·54 (1·40, 1·66)

* The models included a fixed effect for year of intervention, residential distance to the nearest primary health center and baseline population of the cluster.

Supplementary Table 9. The participant-average treatment effect of the intervention (compared to control) and the effect of the intervention period (compared to baseline).

	RR (95% CI) *	
**Outcomes**	**Intervention/Control**[!]	**Study years/Baseline**
Received any antenatal care	1·05 (1·01, 1·08)	1·72 (1·53, 1.95)
Four or more total antenatal visits	1·27 (1·10, 1·46)	2·49 (2·18, 2·83)
Antenatal care initiated in the 1st trimester	1·11 (1·03, 1·20)	1·14 (1·04, 1·24)
Intermittent preventive treatment of pregnancy	1·07 (0·98, 1·16)	2.92 (2·46, 3·47)
Institutional delivery	1·07 (0·93, 1·23)	1·39 (1·25, 1·55)

* The models included a fixed effect for year of intervention, residential distance to the nearest primary health center and baseline population of the cluster.
! Includes year 2 and year 3

Kayentao K, *et al. BMJ Global Health* 2023; 8:e011071. doi: 10.1136/bmjgh-2022-011071
